# Cytotoxic T cell responses are enhanced by antigen design involving the presentation of MUC1 peptide on cholera toxin B subunit

**DOI:** 10.18632/oncotarget.5307

**Published:** 2015-09-21

**Authors:** Wuguang Lu, Lingchong Qiu, Zhanpeng Yan, Zhibing Lin, Meng Cao, Chunping Hu, Zhigang Wang, Jin Wang, Ye Yu, Xiaoyang Cheng, Peng Cao, Rongxiu Li

**Affiliations:** ^1^ State Key Laboratory of Microbial Metabolism, School of Life Sciences & Biotechnology, Shanghai Jiao Tong University, Shanghai, 200240, China; ^2^ Laboratory of Cellular and Molecular Biology, Hospital of Integrated Traditional Chinese and Western Medicine, Nanjing University of Chinese Medicine, Nanjing 210028, China; ^3^ Institute of Medical Science and Department of Pharmacology and Physiology, Shanghai Jiaotong University School of Medicine, Shanghai 200025, China; ^4^ Engineering Research Center of Cell & Therapeutic Antibody, Ministry of Education, School of Pharmacy, Shanghai Jiao Tong University, Shanghai, 200240, China; ^5^ Jiangsu Province Academy of Traditional Chinese Medicine, Nanjing 210028, China

**Keywords:** immunotherapy, MUC1, antigen design, cytotoxic T cell response, vaccine

## Abstract

Induction of cytotoxic T lymphocytes (CTL) is critical to cancer vaccine based immunotherapy. Efforts to elicit CTLs against tumor MUC1 with peptide based vaccine have not been successful in clinical application. We have design a MUC1 vaccine by replacing B cell epitope of CTB with MUC1 VNTR peptide. Immunization with hybrid CTB-MUC1 plus aluminum hydroxide and CpG adujuvant (CTB-MUC1-Alum-CpG) induce MUC1-specific CTLs in mice. Moreover, this vaccination can prevent tumor growth and reduce tumor burden in MUC1^+^B16 mice model. Meanwhile, CTB-MUC1-Alum-CpG vaccination can promote Th1 cells and CD8+ T cells inflate to tumor tissue. Our approach might be applicable to other cancer vaccine design.

## INTRODUCTION

CTLs is critical to the anti-tumor immune response [1]. In therapeutic mouse tumor models, depletion of CTLs(CD8+ T cells) abolish both the tumor immune response and therapeutic efficacy [2]. Furthermore, CTLs having much better tumor target specificity and longevity than other antitumor responses. In addition, CTLs have been the important evaluation index of cancer vaccine and many preclinical and clinical studies showed that strength of CTL correlates with efficacy of cancer vaccine [3–5]. Several attempts have been made to develop cancer vaccines specific binding tumor-associated antigens, including MAGEs, CD20, CTLA-4, and MUC1 [6–8], with the goal of inducing antigen-specific CTLs for cancer therapy.

Mature MUC1 consists of a large extracellular N-terminal subunit and a C-terminal subunit that resides on the cell surface as a heterodimeric complex via strong non-covalent binding [9, 10]. The N-terminal subunit contains a variable number (20 to 125 depending on individual alleles) of 20-amino acid tandem repeats (VNTRs) [10, 11]. Each VNTR is composed of 20 amino acids (APDTRPAPGSTAPPAHGVTS), in which the APDTR segment is the most important antigenic epitope recognized by anti-mucin mAbs and cytotoxic T cells [12]. Various vaccination strategies and immunotherapies targeting the MUC1 N-terminal VNTR have been developed to treat MUC1-positive cancers; many of these have proven capability of inhibiting tumor growth, reducing metastasis, and prolonging survival in MUC1 transgenic mice [13–15].

An approach using synthetic MUC1 VNTR peptides of various lengths, conjugated to carriers, such as keyhole limpet hemocyanin(KLH), bovine serum albumin (BSA), and diphtheria toxin(DT) [16–18] failed to induce effective antitumor responses, especially tumor-specific CTLs [18]. This weak immunogenicity may due to the conformational disparities between the conjugated peptide and that of tumor-expressed MUC1 [13].

Epitope insertion into carrier proteins was proved to be an efficient approach to vaccine design [19–21], due to the facts that the comformation of the inserted peptide is more stable and more close to its native form, compared with N-terminal or C-terminal fusion counterparts [22, 23]. Furthermore, The hybrid protein can be produced in pure form for easier quality control and functional analysis, while almost all chemical conjunction products are mixtures.

Cholera toxin (CT) consists of subunit A (CT-A) and a pentamer of subunit B (CTB). CTB traps mucosal lymphocytes or macrophages or both [24, 25], and lowers the threshold concentration of the conjugated antigen required for immune cell activation; therefore, CTB is a good antigen carrier to stimulate the mucosal immune response [25].

In order to utilize the modulating efficacy of CTB, while avoiding the deleterious effect of carrier epitope-specific suppression on target peptide, we replaced the predicted B cell epitope of CTB (Q_56_–D_59_) with a 12-mer MUC1 VNTR peptide to create a CTB-presented MUC1 antigen. This antigen was expressed by *Escherichia coli* in periplasm, purified, and formulated with aluminum hydroxide gel (Alum) in combination with CpG ODN.

Production of IgG2a-type antibodies reflects the involvement of Th1-type cytokines. Therefore, higher IgG2a/IgG1 ratio points toward Th1-type of immune response. However, IgG2a isotype could not be measured, since the gene that encodes IgG2a is deleted in C57BL/6 mice [26]. Instead, C57BL/6 mice produce antibodies of the IgG2c isotype [27]. Anti-serum analysis indicated that the addition of CpG enhanced the anti-MUC1 IgG2c response and the ratio of IgG2c to IgG1, which is associated with the Th1 response. The cellular immunological responses and protection from tumor challenge exhibited by this CpG-containing formulation could induce MUC1-specific CTLs and cause growth inhibition of MUC1-expressing tumors. Furthermore, this CTB-MUC1-alum-CpG formulation can promote the tumor inflating of T cells, especially CD8+ T cells and Th1 cells. In addition, in therapeutic mice model, CTB-MUC1 significantly reduce tumor burden.

## RESULTS

### The predicted B cell epitopes of CTB

CTB has immunomodulatory effects and is a well-suited antigen carrier to stimulate the mucosal immune response. To find the best MUC1 peptide insertion position, five kinds of epitope prediction methods based on protein amino acid scale and 3D structure were employed to predict the CTB B cell epitopes and the top 5 predicted epitopes of each method are shown in Supplementary table 1. The best B epitopes of CTB were primarily located in the V_50_–A_70_ and A_70_–N_103_ regions. In particular, V_52_–A_59_, located in a loop on the exposed surface of pentameric CTB, is the consensus epitope from all five epitope prediction methods. Whereas E_51_–S_55_ is thought to prevent pentamer formation [28], Q_56_–D_59_ might be the most antigenic epitope for replacement with and presentation of the MUC1 peptide conformation.

### Homology model and structural stability of hybrid CTB-MUC1

**Figure 1:**
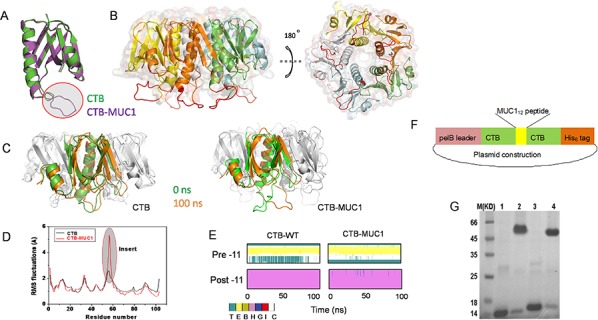
Homology modeling, MD simulation, and construction of CTB and hybrid CTB-MUC1 presentation **A.** Structure comparison of monomer CTB-MUC1 to CTB. The red cycled purple loop is the replaced 12-mer MUC1 peptide. **B.** Structure comparison of pentameric hybrid CTB-MUC1 to CTB. The red loops floating on the protein surface represent the presented MUC1 peptide. **C.** Structure comparison of 100 ns to 0 ns MD simulation: left, CTB monomer in CTB pentamer; right, CTB-MUC1 monomer in CTB-MUC1 pentamer. The brown cartoon structure is 100 ns, green is 0 ns. **D.** RMSF analysis of CTB and CTB-MUC1. **E.** Secondary structure analysis of CTB and CTB-MUC1 in 100 ns MD simulations. Pre-11 is the 11 amino acids adjacent to the N terminus of the replaced MUC1 peptide. Post-11 is the 11 amino acids adjacent to the C terminal of the replaced MUC1 peptide. **F.** Construction of His_6_-tagged CTB-MUC1expression vector. **G.** SDS-PAGE analyses of the production of recombinant CTB and CTB-MUC1 pentamer. Lane 1: CTB monomer; Lane 2: CTB pentamer; Lane 3: CTB-MUC1 monomer; Lane 4 CTB-MUC1 pentamer. To detect the pentameric CTB and CTB-MUC1, the purified proteins were mixed with 2 × non-reducing sample buffer and directly loaded onto the gel without heating.

The homology model of hybrid CTB-MUC1 fusion protein was constructed based on the X-ray structure of the CTB pentamer. The homology modeling results suggested that the insertion of the MUC1_12_ peptide did not disturb the skeleton structure of the CTB carrier. The inserted MUC1_12_ peptide presented as a loop floating on the surface of pentameric CTB-MUC1 fusion protein (Figure 1A, 1B). The 100-ns MD simulations of CTB and CTB-MUC1 suggested that the CTB-MUC1 pentamer has stability similar to that of pentameric CTB (Figure 1C). Root-mean-square fluctuation (RMSF) analysis showed that the whole protein elicited similar residual fundamental mobility except the insertion (Figure 1D). Moreover, analysis of the secondary structure of 11 amino acids on either side of the insertion indicated that the presence of the MUC1 peptide loop did not disturb the secondary structure of CTB (Figure 1E). In addition, the comparison of all insertion positions showed that among the four insertions, MUC1 at Q_56_–D_59_ insertion site adopt a conformation more close to native one(Supplementary figure 1).

### Production of hybrid CTB-MUC1

Hybrid CTB-MUC1 protein was constructed by displacement and insertional mutagenesis (as described in Materials and Methods), and expressed in *E. coli* (TG1) cells. The construction of the expression vector is shown in Figure 1F. Consistent with the modeling and simulation results, the recombinant protein expressed in *E. coli* was soluble, and formed a pentamer (Figure 1G). Approximately 25 mg CTB-MUC1 fusion protein (90% pure) can be obtained from 1 liter of bacterial culture. The hybrid CTB-irrel also can formed a pentamer while CTB conjugated MUC1 was a heterogeneous mixture (Supplementary Figure 2).

### Serological responses

**Figure 2:**
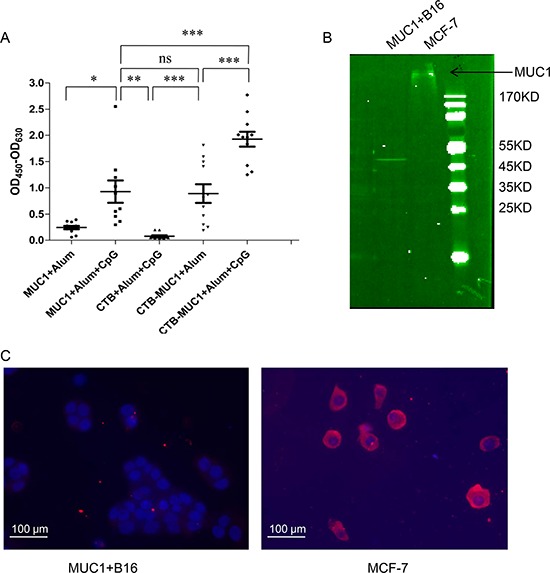
Antibody specificity analysis by ELISA, Immunoblotting and immunofluorescence **A.** Results shown represent anti-MUC1_30_ peptide responses from pooled mouse sera obtained 7 days after the second immunization, as assessed by ELISA. **B.** Immunoblots of membrane protein from MUC1+ B16 cells and MCF-7 with anti-MUC1 antibody. Each data point represents an individual mouse and the horizontal lines indicate the mean for the group of mice(*r* = 3, *n* = 10). **C.** The fluorescence images are shown the tumor cell surface binding of anti-serum form CTB-MUC1-Alum-CpG ODN group.

As shown in Figure 2A, Mice intra-nasally immunized with CTB plus alum or alum-CpG adjuvant, showed no detectable anti-MUC1 responses. In contrast, mice immunized with MUC1_12_ peptide plus alum or alum-CpG, CTB-MUC1 protein plus alum or alum-CpG elicited robust anti-MUC1 antibody responses with alum-CpG group induced significantly higher anti-MUC1 antibody response. Titer measurements of IgG subtype (Supplementary table 2) showed that the CTB-MUC1-alum-CpG group elicited higher IgG2c responses and higher ratio of IgG2c/IgG1, indicating Th1 polarization.

MUC1^+^B16 cells expressed recombinant MUC1 VNTR-GFP fusion protein, which is about 30 KD [29]. As shown in Figure 2B and 2C, MUC1+B16 cells expressed no MUC1 on cell surface while MCF-7 expressed full length MUC1 on the cell surface. Furthermore, anti-serum from CTB-MUC1-Alum-CpG ODN group can recognize and bind native MUC1 on MCF-7 cells(Figure 2C). These results also suggested that MUC1^+^B16 tumor cell only intracellular expressed partial MUC1.

### Tumor protection efficacy

**Figure 3:**
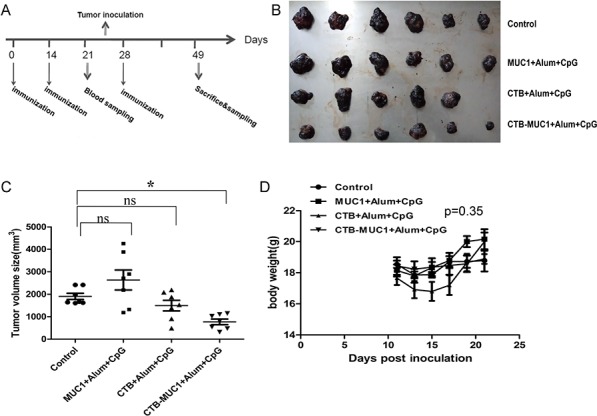
Tumor protective efficacy of CTB-MUC1 on MUC1^+^B16 melanoma-bearing mice Tumor volume change was determined every 2 days during the delivery period, and healthy mice were used as normal controls in this study. The values are expressed as the mean volume of tumors ± SD. **A.** Vaccination schedule. Mice got 3 immunizations on day 0, day 14 and day 28. Mice after 2 immunizations were given inoculations with MUC1^+^B16 cells on day 24. **B.** Tumors were removed from mice in different groups and photographed. **C.** Volume statistics of separated tumors after sacrifice. Each data point represents an individual mouse and the horizontal lines indicate the mean for the group of mice (*n* = 6–10). **D.** The weight change for each mouse was determined every 2 days during the delivery period. Statistical differences were analyzed by Student's *t*-test.

To evaluate the tumor growth inhibition activity of CTB-MUC1 immunization *in vivo*, four groups of mice immunized with CpG formulation were challenged with MUC1^+^ B16 tumor cells on day 24(Figure 3A). On day 49, all mice were sacrificed and dissected to measure tumor volume and obtain tumor mass. As shown in Figure 3B and 3C, CTB-MUC1-alum-CpG vaccination significantly reduce tumor burden while the other 3 groups showed no tumor inhibitory activity. Mice body weights were measured during administration to assess the effect of vaccination and tumor bearing on quality of life. No significant weight loss was observed in mice treated with the four different formulations (Figure 3D).

### Effect of CTB-MUC1 immunization on T lymphocyte responses

**Figure 4:**
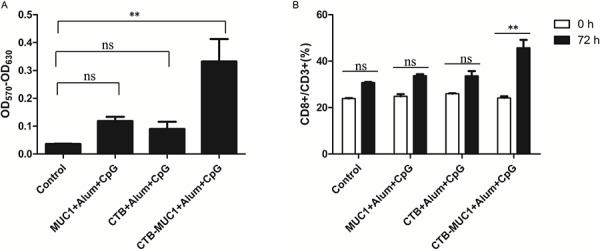
T lymphocyte proliferation assay 7 days after the second immunization(day 21), splenic lymphocytes from mice(groups containing CpG) were cultured with 10 μg/ml of MUC1_12_ peptide. After 72 h, cell viability was measured by MTT assay **A, B.** Statistics relating to percentage of CD8^+^T cells among CD3^+^ T cells after simulation detected by flow cytometry. Each data point represents an individual mouse, and the horizontal lines indicate the mean± SD for the group of mice(*r* = 3, *n* = 3).

We stimulate splenocytes from mice of different vaccinated groups with Synthesized MUC1_12_ peptide for 72 hours to examined the celluar proliferation. As shown in Figure 4A, cell prolliferation was only observed in the group immunized with CTB-MUC1-alum-CpG while splenocytes from physiological saline (control), MUC1-alum-CpG and CTB-alum-CpG group showed no celluar viability differences, (*p* < 0.05; Figure 4A). The percentage of CD8^+^ lymphocytes in total lymphocytes (CD3^+^) was further detected by flow cytometry. After MUC1_12_ peptide simulation, the percentage of CD8^+^ lymphocytes from CTB-MUC1-alum-CpG group increased 2 folds while other groups was nearly no change. 3 mice were randomly picked from each group and each sample replicate for 3 times for the CTL assay and statistics (*n* = 3, *r* = 3).

**Figure 5:**
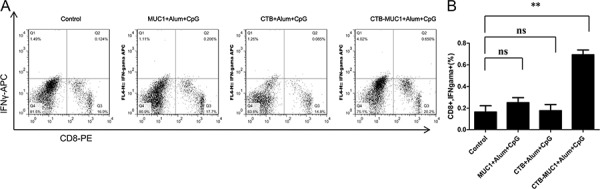
IFNγproduction of CD8^+^ T cells stimulated with MUC1_12_ peptide Splenic lymphocytes were isolated after sacrifice and stimulated with MUC1_12_ peptidefor 72 h, and BFA was added into the cells to block IFNγ secretion. After stimulation, intracellular IFNγwas analyzed by flow cytometry using APC-anti-IFNγand PE-anti-CD8. Frequencies of IFNγ-producing cells inactivated T-cell fractions gated with CD8 are shown as percentages **A, B.** Statistics relating to the percentage of IFNγ^+^ cells among CD8^+^ cells. Each data point represents an individual mouse and thehorizontal lines indicate the mean± SD for the group of mice(*r* = 3, *n* = 3).

The antigen-specific T cell responses from all immunization groups were analyzed through IFNγ production by T lymphocytes upon stimulation with MUC1_12_ peptide *in vitro*. As shown in Figure 5A, after MUC1_12_ peptide simulation, the percentage of CD8^+^ IFNγ^+^ lymphocytes from CTB-MUC1-alum-CpG group increased 5 folds while other groups was nearly no change compare to control. Figure 5B demonstrates IFNγ staining and statistics in 3 randomly picked mice and repeat for 3 times(*n* = 3, *r* = 3).

### Cytotoxic T-lymphocyte responses

**Figure 6:**
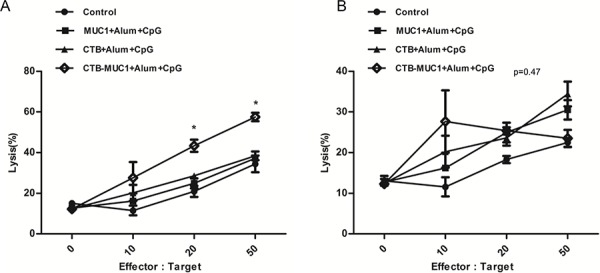
The degree of cytotoxicity of MUC1 peptide-stimulated splenic lymphocytes (effector cells) in mice from three groups immunized with the formulation containing CpG and normal control mice on MUC1^+^ B16 cells **A.** and G418-resistant B16 cells **B.** (target cells) depends on the ratio of splenic lymphocytes to MUC1^+^ B16 cells (Effector:Target). MUC1^+^ B16 cells were labeled with CFSE and co-cultured with MUC1 peptide-stimulated splenic lymphocytes at the ratios indicated above. When the ratios was “0”, it means “only target cells”. Cells were co-cultured for 4 h at 37°C. At the end of the experiment, dead cells were labeled with 7-AAD. The percentage of MUC1^+^ B16 cell lysis at different Effector:Target ratios was analyzed by a flow cytometer (*r* = 3, *n* = 3).

MUC1^+^B16 cells were labeled with CFSE and co-cultured with effector cells at the serial ratios. After co-culture for 4 h, dead cells were labeled with 7-AAD. The percentage of dead MUC1^+^B16 cells was analyzed using flow cytometry. As shown in Figure 6 A, at a 50:1 ratio of effector cells to target cells, 59.6% of MUC1^+^B16 cells were dead in CTB-MUC1-alum-CpG formulation-immunized mice, whereas the percentage of target cell death was only 30% in other groups. The CTB-MUC1 group elicited higher cytotoxicity even at the lower ratio of 10:1, the percentage of target cell death was 16–25%, and there was no significant difference between the groups. However, when G418-resistant B16 cells were used as target cells, it exhibited erratic changes in cell death at different Effector: Target ratios. 3 mice were randomly picked from each group and each sample replicate for 3 times for the CTL assay and statistics (*n* = 3, *r* = 3 *p* < 0.05).

### Detection of tumor-infiltrating lymphocytes and MUC1 expression in tumor tissues

Lymphocyte infiltration into tumors is relevant to prognosis in cancer treatment. We used a qPCR assay to detect tumor-infiltrating lymphocytes, Th1 responses, and MUC1 expression in tumor tissues from different immunized groups. Mouse T cell surface marker CD3 epsilon chain (CD3e) and CD8 alpha chain (CD8a) were chosen as test criteria for tumor-infiltrating lymphocytes, while IFNγRβ, CCR2, IL-2Rα and CCR5 were chosen as Th1-associated markers. MUC1 expression was significantly reduced in tumors from mice vaccinated with the CTB-MUC1-alum-CpG formulation (Supplementary figure 3A). Moreover, the mRNA levels of CD3e and CD8a were increased about 10-fold and 15-fold, respectively, in tumors from mice vaccinated with the CTB-MUC1-alum-CpG formulation (Supplementary figure 3B, 3C). Moreover, after vaccination with the CTB-MUC1-alum-CpG formulation, the mRNA level of Th1-associated markers IFNγRβ, CCR2, IL-2Rα and CCR5 also increased about 5-fold compared to the control (Supplementary figure 3D, 3E, 3F, 3G). These results suggest that there was significant infiltration of T cells and Th1 cells in tumors from mice vaccinated with the CTB-MUC1-alum-CpG formulation.

### Tumor burden reduction in therapeutic model

To assess the therapeutic effects, vaccine was administered after tumor inoculation. First vaccination was given when mice tumor incidence reach 90%(about 7 days) and one dose per week(Supplementary figure 4A). After 3 dose vaccinations, CTB-MUC1-alum-CpG formulation significantly inhibit tumor growth compared with mice given CTB-irrel-CpG (Pep-irrel) or CTB∞MUC1-alum-CpG formulation (Supplementary figure 4B, 4C). However, no significant weight change was observed in mice treated with different formulations (Supplementary figure 4D).

## DISCUSSION

Antigen-specific CD8^+^ CTLs play a pivotal role in antitumor immunity. However, vaccines containing conjugated or unconjugated MUC1 peptides, plus various adjuvants, failed to induce therapeutic tumor-specific CTLs *in vivo* [17], which may be due to protein conformational dissimilarities, improper vaccine formulations, and inadequate adjuvants [13, 15]. To solve this problem, multiple approaches have been developed to generate protein vaccines with high CTL induction activity, such as recombinant epitope vaccine, carrier-conjugated peptide vaccine, and protein antigen combined with various adjuvants that can enhance Th1 responses. Among these approaches, a MUC1 peptide corresponding to five tandem repeats has been found to have the capacity for CTL induction [30], and MUC1_90_-KLH showed better protection from tumor challenge than that of MUC1_32_-KLH [17], suggesting that the conformation of peptide maybe more close to the native form when the size of peptide is increased.

CTB forms a non-covalent pentamer and binds to the TLR receptor and activate mucosal immunity robustly, making it a good carrier protein or adjuvant in vaccine development [31]. However, simply conjugate MUC1 peptide to CTB plus CpG ODN as adjuvant was unable to induce MUC1-specific CTLs in MUC1 Tg mice or C57BL/6 mice [25]. In this study, we took a different approach to keep the conformation of MUC1 peptide in native form by inserting the MUC1 peptide into the internal side of CTB. We choose a B cell epitope of CTB as insert site to hoping to balance the immune response more towards MUC1 than CTB.

First, the most probable B cell epitope on the carrier protein CTB was predicted using bioinformatic tools, and the consensus best B cell epitope was replaced with a 12-mer MUC1 peptide. We choose the epitope site which is on the surface of the CTB and dose not interfere the pentamer formation of CTB. To confirm in silico, we employ homology modeling, and molecular dynamics simulation tools. Indeed, the result indicated that the comformation of MUC1 peptide in hybrid CTB-MUC1 formed a flexiable loop which is more close to the native form of MUC1 than N-terminal or C-terminal fusion. Our data demonstrate that this kind of MUC1 peptide displacement/insertion in the CTB pentamer can induce MUC1-specific CTLs result in MUC1^+^ B16 tumor protective efficacy and therapeutic efficacy. In contrast, CTB conjugated MUC1 have no therapeutic efficacy in MUC1^+^ B16 tumor bearing mice.

Alum salts are thought to be a good vehicle to controlled released the adsorbed antigen in tissues [32]. CpG DNA is a novel adjuvant that is known to promote Th1-type immune responses with the secretion of IFNγ, TNFα and IL-12 cytokines, opsonizing antibodies, such as those of the IgG2a(IgG2c in C57BL/6 mice) isotype, and strong CTL induction [33]. Combination CpG DNA+alum had the greatest potential to augment immune responses with minimal side effects and gave mixed Th1/Th2 responses [33]. In this study, the vaccine antigen design of conformationally strained CTB-MUC1 presentation, formulated with CpG ODN plus alum, formed a CTB-MUC1-alum-CpG complex(data not shown) and induced a stronger anti-MUC1 IgG antibody response and Th1-specific IgG2a(IgG2c in C57BL/6) antibody response than aluminum hydroxide gel alone. In addition, 0.1 mg Alum(calculate from 2% Aluminium hydroxide gel, Invtrogen US)) can absorb at least 0.5 mg CTB-MUC1 proteins and 50 μg CpG ODN 1826(data not shown). This result confirmed the effects of CpG ODN on vaccination, and the addition of alum is particularly desirable, because it can induce stronger cellular immunity than alum alone [33]. Furthermore, the antibody produced from the vaccination of CTB-MUC1-alum-CpG can specially recognize and bind natural human MUC1 on MCF7 cells. However, our data indicated that MUC1^+^B16 tumor cell manly intracellular express MUC1 but not the on-membrane expression. As a result, the anti-tumor effect of CTB-MUC1 was impossibly come form antibody-dependent ADCC.

Previous studies have revealed that one of the factors that limit the efficacy of T-cell-based cancer immunotherapy is T cell access to the tumor [34]. Tumor infiltration by T cells, especially Th1 and CD8^+^ T cells, correlates with favorable outcome and prolonged patient survival [34, 35]. Analysis of mRNA levels of CD3e, CD8a, and the Th1-associated markers IFNγRβ, CCR2, IL-2Rα and CCR5 in tumors from differently immunized groups revealed that immunization with the CTB-MUC1-alum-CpG formulation led to significantly increased infiltration of T cells, especially CD8^+^ T cells, and Th1 cell clone proliferation in tumor tissues.

In conclusion, the study presented here demonstrates that our design can activate MUC1-specific CTLs result in tumor suppression in mice. Moreover, the bioinformatics tools, especially computer simulation, are very good approaches for peptide vaccine design. A rational designed peptide vaccine may induce strong antigen-specific immune responses and tumor-suppression efficacy.

## MATERIALS AND METHODS

### Peptides, plasmids, and strains

Two MUC1 peptides were synthesized by GeneScript(Nanjing, China)with the sequences: NH2-VTSAPDTRPAPGSTAPPAHGVTSAPDTRPA-COOH(MUC1_30_) and NH2-APDTRPAPGSTA-COOH(MUC1_12_). *E. coli* strain Top10, TG1, and a CTB gene, containing the periplasmic expression vector pCTB2, were prepared in our lab.

### Cell line, animals, and adjuvants

MUC1^+^ B16 cells stably expressing human MUC1 VNTR(fused with GFP) were a kind gift from the Laboratory of Molecular Biology, College of Basic Medical Science, Jilin University. B16 cell stable transected with pCDNA3 plasmid (G418-resistant B16 cell)was prepared in our lab. These two cell lines are constructed as described [29]. Female C57BL/6 mice were purchased from the Chinese Academy of Sciences, Shanghai Experimental Animal Center and housed on a 12-h light/dark cycle with food and water under specific pathogen-free conditions, according to the guidelines of the Association for Assessment and Accreditation of Laboratory Animal Care International. All *in vivo* studies were carried out under approved institutional experimental animal care and protocols. Adjuvant aluminum hydroxide gel(alum) was purchased from Invitrogen (Invitrogen US). A mouse-specific CpG oligo deoxynucleotide (CpG ODN 1826, TCCATGACGTTCCTGACGTT) was synthesized by GeneScript (Nanjing, China).

### Prediction of B cell epitope in CTB

The B cell epitope was predicted using the online prediction tool Immune Epitope Database(IEDB, http://www.iedb.org/). Several prediction methods were used to analyze continuous B cell epitopes in CTB, including Chou and Farman'sturn scale, Bepipred Linear Epitope Prediction, Emini Surface Accessibility Prediction& Discotope.

### MUC1 peptide insertion, homology modeling, and molecular dynamics

Amino acids Q_56_–D_59_ of CTB were replaced by a 12-aa MUC1 peptide (NH2-APDTRPAPGSTA-COOH) using Discovery studio 3.5. A homology model of the CTB-MUC1 fusion protein was built based on the X-ray structure of the CTB pentamer(PDB entry 1FGB, 2.4Å) and human MUC1 peptide(PDB entry 1SM3, chain P, 1.95Å) using Modeller9V7. The constructed model was checked and validated by the program Procheck [36]. MD simulations were performed for 100 ns according to our previous procedure using DESMOND 3.0 with default settings [37]. All structure maps were produced using pMOL 1.5 software [38]. To compare the influence of different insertion positions on CTB-MUC1 structure, the other three best epitope P_2_-I_5_, A_32_-R_35_ and N_90_-P_93_ from different part of CTB were also replaced by MUC1 peptide and analyzed by homology modeling and molecular dynamics simulation as described above.

### Expression and purification of hybrid CTB-MUC1

pCTB2, a periplasmic expression vector containing the CTB gene was used as the source of CTB DNA. Insertion mutagenesis was performed to produce hybrid CTB-MUC1 protein, as follows. The pCTB2 vector was amplified by polymerase chain reaction(PCR) using KOD plus polymerase (Toyobo, Japan) and the following primers: 5′-AATTTTTCAAGTAGA AGTACCAGGTAGT**GCTCCGGACACCCGTCCGGC TCCGGGTTCTACCGCT**TCACAAAAAAAAGCGA TTGAAAGG-3′ (forward) and5′-CCTTTCAATCGCTTTTTTTTGTGA**AGCGGTAGAACCCGGAGCCGGACGGGTGTCCGGAGC**ACTACCTGGTACTTCTACTTGAAAAATT-3′ (reverse) (the sequence encoding the NH2-APDTRPAPGSTA-COOH peptideis in bold). Methylated parental DNA was digested with Dpn I and the PCR product was transformed into *E. coli* TOP-10 competent cells. The sequences of the mutagenized plasmids were confirmed by automated sequencing. The resulting plasmid was transformed into *E. coli* TG1 for expression. A single colony of the *E. coli* TG1 strain containing each of the plasmids described above was inoculated into Luria-Bertani (LB) medium containing ampicillin (100 μg/ml) and grown at 37°C overnight with rotation at 220 rpm. The next morning, 25 ml of the overnight culture was transferred into 1 liter of fresh M9 medium to permit exponential growth. After about 36 h incubation at 28°C, protein expression was induced by the addition of 0.2 mM IPTG and 100 ml 10 × TB culture media, and cells were allowed to grow at 30°C for another 60 h. Cells were collected by centrifugation, resuspended in 100 ml ice-cold lysis buffer (50 mM Tris-HCl at pH 8.0, 25 mM NaCl, 2 mM EDTA), and lysed by adding 5 ml of freshly prepared lysozyme (100 μg/ml final concentration from 3 mg/ml aqueous solution) followed by incubation at room temperature for 30–50 min with occasional shaking until the suspension became viscous. The lysates were treated with 200 μl of DNase I (Sigma) (15 units/μl stock in 1 M MgCl_2_) and incubated at room temperature for an additional 20–30 min until the suspension became watery. Cellular lysates were centrifuged at 12,000 rpm for 15 min at 4°C and filtered through a 0.45-μm filter. Purification of His_6_-tagged CTB and CTB-MUC1 was carried out on an AKTA-purifier 900 system (Amersham Biosiences, Sweden). The supernatant was dialyzed into 1 × PBS, pH 7.4 and loaded onto an His-Trap HP column (5 ml; GE Healthcare, UK) pre-equilibrated with buffer A(1 × PBS, pH 7.4). The protein of interest was eluted with a non-continuous gradient to 100% of buffer B(1 × PBS, pH 7.4, 500 mM imidazole). The pooled eluate containing the target protein(typically a sharp high elution peak) was dialyzed against 1 × PBS, pH 7.4, 5% glycerol for 12 h at 4°C, and then the dialyzed buffer was changed to 1 × PBS for another 12 h to remove residual imidazole. To produce a hybrid non-specific peptide control, a 12 mer peptide(DSTSSPVAHSGT) from mouse MUC1 VNTR was construct as described above and the resulted product was named CTB-irrel.

The conjugation of CTB and MUC1 was performed by *m*-maleimidobenzoic acid N-hydroxysuccinimide ester(MBS) conjugation method as described [39]. Briefly, 1 mg MBS was dissolved in 100 μL dimethylformamide (DMF) and then mixed with 5 mg CTB (5 mg/ml in PBS) and incubated for 30 min at room tempreture to activate CTB. After desalting, about 3 mg MBS activated CTB was mixed with 3 mg MUC1 peptide(dissolved in 100 μL DMF) and incubated for 12 h with rotating at 4°C. The resulted conjugation product was named CTB∞MUC1.

### Vaccine preparation and immunization

For injection-hypodermatica (IH) immunization, mice were immunized with 0.4 ml of antigen (MUC1, CTB-MUC1, 50 μg per dose)in combination with aluminum hydroxide (0.3 mg per dose), and antigen (MUC1, CTB, CTB-MUC1, 50 μg per dose) in combination with aluminum hydroxide (0.3 mg per dose) and CpG ODN (30 μg per dose). Vaccinations were done on days 0, 14, and 28. Serum samples for serological assay were collected on day 21.

### Serological assays

Antibody titers were determined by an enzyme-linked immunosorbant assay(ELISA) as recently described [13]. ELISA plates were coated overnight at 4°C with 0.1 μg MUC1_30_ peptide or recombinant CTB protein and blocked with 3% BSA in 1 × PBS. Serum samples were diluted in 1 × PBS (with 3% BSA, dilution ratio: 1:100). After incubation with serum dilution at room temperature for 2 h, The plates were washed and incubated with rabbit anti-mouse IgG-HRP antibodyfor 2 h at room temperature. The plates were washed and developed with substrate 3,3′,5,5′-tetramethylbenzidine (TMB). The absorbance was read at two wavelengths (405 and 490 nm). For antibody titer detection, serum samples were serially diluted 2-fold from the initial 100-fold dilution. Titers are defined as the highest dilution yielding an optical density of 0.1 or greater relative to normal control mouse sera.

### Western blottig & Immunofluorescence staining

To investigate MUC1 expression on the surface of MUC1+B16 cell, MCF7 and MUC1+B16 cell surface protein were isolated using Cell Surface Protein Isolation Kit(Pierce) according to the standard manual. The isolated cell surface protein samples were analyzed by western blotting. Samples were separated by SDS-PAGE and transferred to PVDF membrane. The membrane was incubated overnight at 4°C with anti-MUC1 antibody(abcam, ab22711), followed by FITC-flourecent goat anti mouse IgG and finally visualized using Odyssey fluorescence imaging system(LICOR, US). To further investigate the binding profiles of anti-serum from the CTB-MUC1-alum-CpG group with native human MUC1, surface plasmon resonance analysis was conducted as previously described [40]. cells were blocked in phosphate-buffered saline (PBS) supplemented with 5% BSA and then incubated at 37°C with anti-serum from the CTB-MUC1-alum-CpG group. (1:100) for 1 h. The cells were then washed three times with PBS (pH 7.4) and then incubated at 37°C with PE-labeled rat anti-mouse IgG1 for another 1 h and fixed before labeling the nuclei with Hoechst 33258. Next, the cells were observed with a flouresent microscope (Zeiss).

### Tumor challenge

Based on the serological assay results, immunized groups containing CpG were identified for tumor challenge and physiological saline was used as vaccine solvent and as normal control. Mice were challenged with MUC1^+^ B16 cells (2 × 10^5^ cells) on day 24, and further immunized on day 28. Palpable tumors were measured by calipers, and tumor volume was calculated as: volume (in grams) = [(length) × (width) ^2^]/2, where length and width are measured in millimeters. On day 49, the mice were sacrificed, and tumors were surgically removed for further analysis.

### T lymphocyte proliferation assay

Cell proliferation was tested using the 3-(4, 5-dimethylthiazol-2-yl)-2, 5-diphenyl tetrazolium bromide (MTT) assay kit according to the standard protocols (Cayman, USA). In brief, lymphocytes were isolated from mouse spleens on day 21, and plated in a 96-well flat bottom tissue culture plate at a concentration of 1 × 10^5^ cells/well in 100 μl of RPMI-1640 culture medium supplemented with 10% fetal calf serum (FCS), and stimulated with 10 μg/ml MUC1_12_ peptide. After 72 h of culture, 10 μl MTT solution was added per well. After 4 h of incubation, colored crystals of formazan were dissolved with 100 μl dimethylsulfoxide(DMSO). Plates were kept on orbital shaker for 5 min and optical density (OD) was read using a multiwell scanning spectrophotometer (ELISA reader) at 570 nm (reference wavelength 630 nm). For T cell subtype detection, freshly isolated spleen lymphocytes were stimulated with MUC1_12_ peptide for 72 h as described above. After washing, cells were stained with anti-mouse CD3-APC and CD8- PE(EB) and analyzed using a FACS Calibur instrument (Becton Dickinson, USA). Unstimulated cells were used as negative control. All experiments were repeated three times and the average results were calculated.

### Intracellular interferon γ (IFNγ) staining

Intracellular staining for IFNγ production was performed as recently described [41]. On day 21, mice were sacrificed, and spleen lymphocytes were isolated and stimulated with MUC1_12_ peptide (10 μg/ml) for 72 h. Six hours before staining, brefeldin A (BFA) was added to the cells to block IFNγ secretion. Cells were stained with anti-Mouse CD8-PE. After washing, cells were fixed with IC fixation buffer (EB) for 30 min at room temperature, permeabilized with permeabilized 1 × EB permeabilization buffer, and stained with anti-mouse IFNγ-APC(antibody dilution: 1:100 in 1 × EB permeabilization buffer), and analyzed using FACS Calibur (Becton Dickinson).

### Cytotoxicity staining

Cell-mediated cytotoxicity was examined using a 7-aminoactinomycin D (7AAD)/carboxyl fluorescein diacetate succinamidyl ester (CFSE) cell-mediated cytotoxicity assay kit (Abnova, USA). On day 21, mice were sacrificed, and spleen lymphocytes were stimulated with 10 μg/ml MUC1_12_ peptide and cultured for 72 h in the presence of 20 units/mL IL-2. Cells were then used as effector cells for the cytotoxicity assay. The flow cytometry-based method was used for cytotoxicity assay. Briefly, CFSE-labeled MUC1^+^ B16 target cells were incubated with effectors at different effector-to-target ratios at 37°C for 4 h. After staining with 7-AAD dye to mark dead or dying cells, cytotoxicity was evaluated by flow cytometry. Cytolysis was determined as the ratio of 7-AAD^+^CFSE^+^ cells to total CFSE^+^ cells. G418-resistant B16 cell was set as negative control.

### Quantity PCR assay

On day 49, total RNA of tumor tissues from sacrificed tumor bearing mice was prepared using TRIzol reagent (Invitrogen). RNA (1 μg) was reverse-transcribed into cDNA using Maxima First Strand cDNA Synthesis Kit according to the standard protocol(Fermentas, USA). qPCR was performed using the 2× Maxima SYBR Green/ROX qPCR Master Mix kit with ABI 7500 according to the standard protocol(Fermentas, USA). Data was analyzed using the ββCT relative quantification method. Primers sequences and corresponding genes are shown in Supplementary table 3.

### Tumor therapy in C57BL/6 mice

For the tumor therapy experiments, C57BL/6 mice (10 per group) were injected subcutaneously with 2 × 10^5^ MUC1^+^ B16 cells per mouse on day 0, and then immunized with antigen (MUC1, CTB, CTB-MUC1, CTB-irrel, CTB∞MUC1, 30 μg per dose) in combination with aluminum hydroxide (0.3 mg per dose) and CpG ODN (30 μg per dose). Vaccinations were done on days 7, 13, and 20. On day 25, mice were sacrificed and tumors were surgically removed and measured weight.

### Statistical analyses

Statistical significance was analyzed with Prism version 5.0, GraphPad Software, Multiple comparisons were performed using one-way ANOVA with Bonferroni's multiple comparison test. Differences were considered significant when *P* < 0.05. Asterisks in figures indicate statistically significant difference (*P < 0.05, **P < 0.01, and ***P < 0.001) and ns indicates no significant difference.

## SUPPLEMENTARY FIGURES AND TABLES


