# Supplementary material for: Cytotoxic T cell responses are enhanced by antigen design involving the presentation of MUC1 peptide on cholera toxin B subunit
Source: Oncotarget. 2015 Sep 21;6(33):34537–48. doi: 10.18632/oncotarget.5307 (PMC4741471; doi:10.18632/oncotarget.5307)
Supplement: Supplementary file 1 [file oncotarget-06-34537-s001.pdf]

SUPPLEMENTARY FIGURES AND TABLES

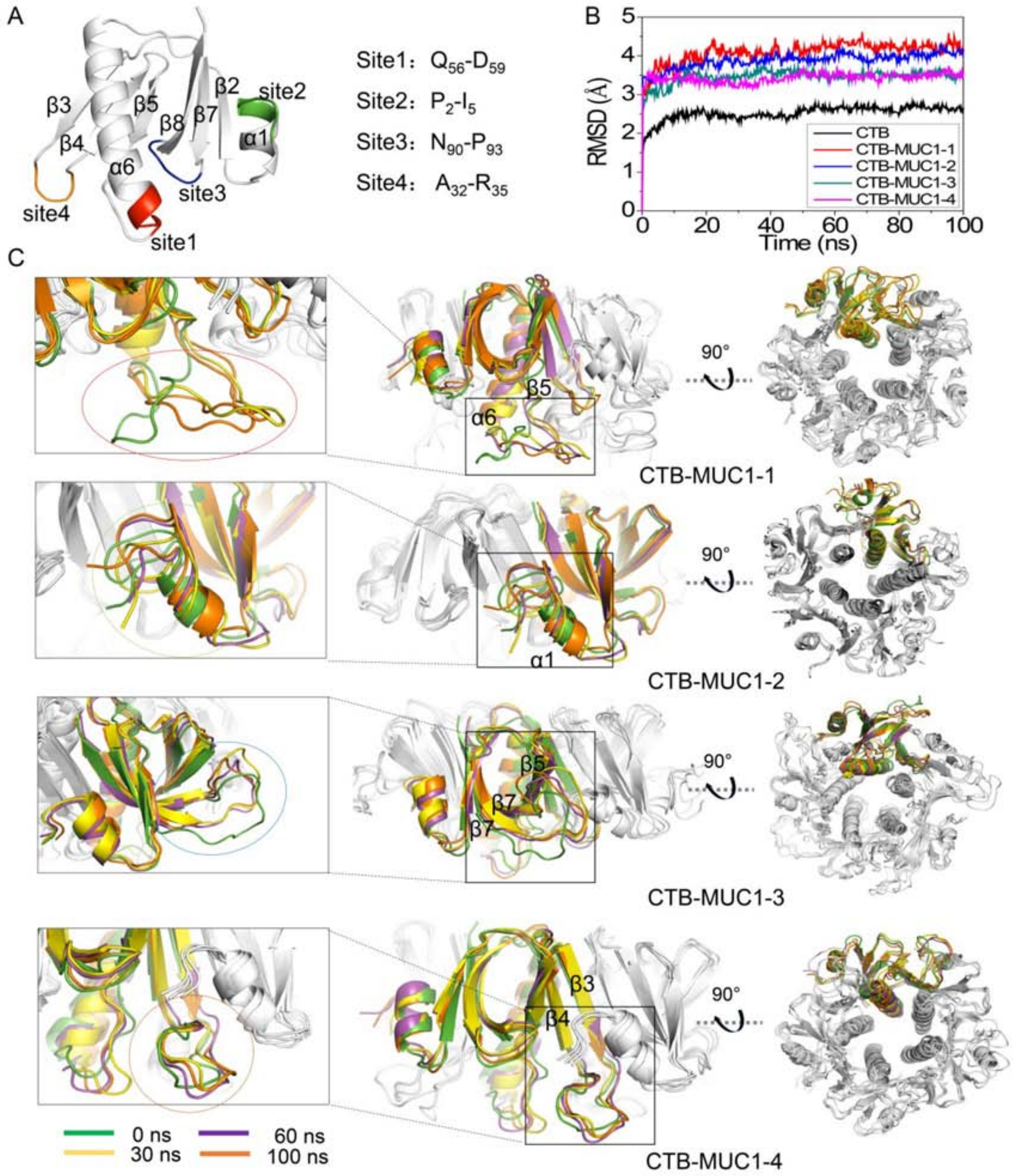

Supplementary Figure S1: The comparison of the conformations of MUC1 in various positions insertion A. The structure of CTB monomer CTB consists of two α-helix and six β-sheet. Site 1 to site 4 showed the insertion position. **B.** The RMSD analysis of four hybrid CTB-MUC1. **C.** The structure comparison of four insertions at 0 ns, 30 ns, 60 n.s, 100 ns MD simulation. The cycles showed the inserted MUC1 at different positions. The homology models of the four insertions quickly reach the steady state after starting the dynamic simulations. However, MUC1 in the P_2-I_5 insertion is interfere with CTB pentamer formation. Moreover, MUC1 in the N_{90}-P_{93} insertion is gradually close to the β5 chain of a CTB submit and sustained interacting with it during dynamic simulation. N_{32}-P_{35} insertion site is between β3 and β4 chain of a CTB submit. MUC1 in this insertion site gradually formed a rigid loop inside the CTB molecular during dynamic simulation, which is also not good for antigen presentation.

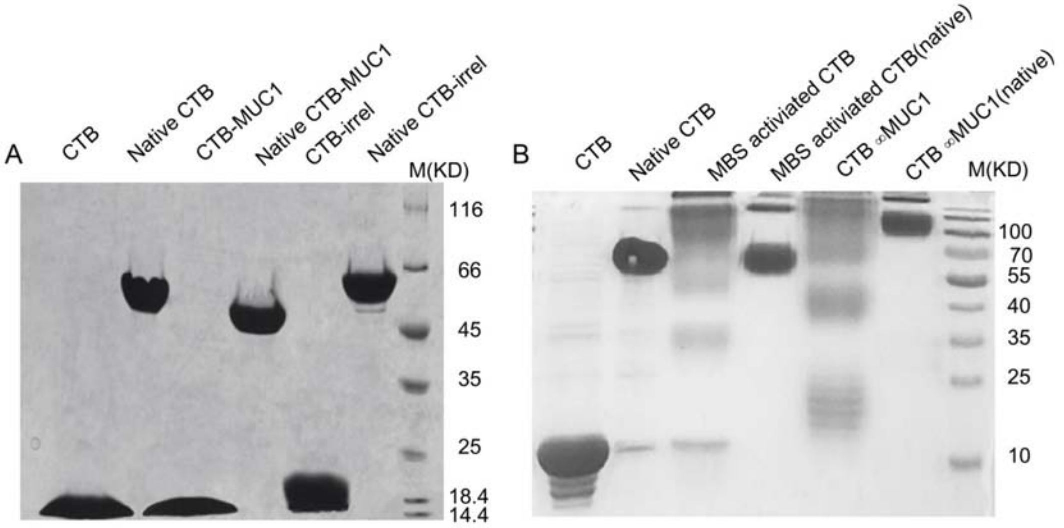

Supplementary Figure S2: SDS-PAGE analysis of production of recombinant CTB-irrel and CTB∞MUC1. A. The purified CTB, CTB-MUC1 and CTB-irrel. **B**. The conjugation of CTB∞MUC1.

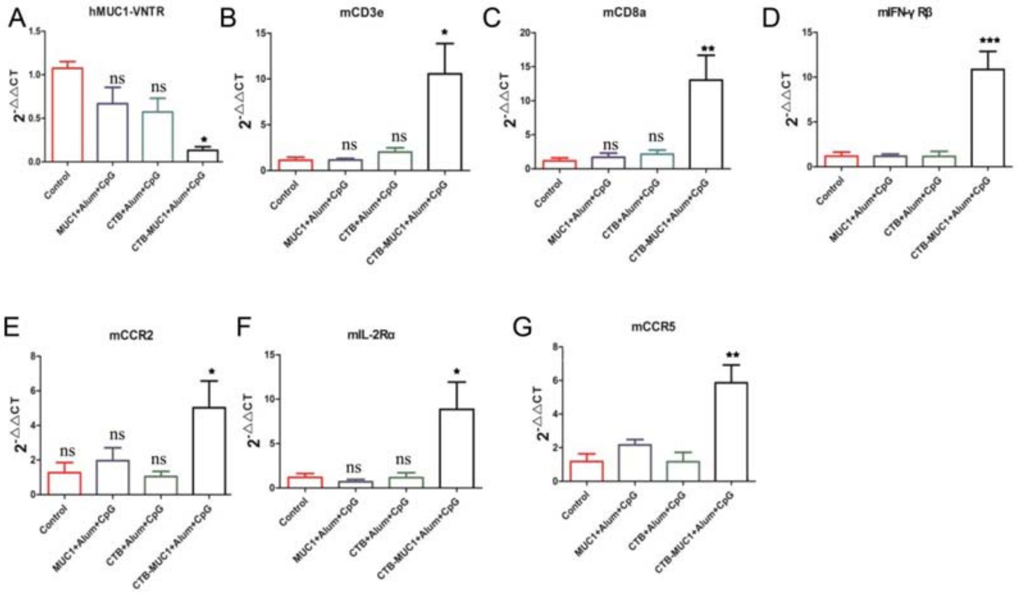

Supplementary Figure S3: mRNA expression of MUC1, CD3e, and CD8a in tumor tissues from sacrificed mice by quantitative reverse transcription-PCR (qRT-PCR). Differences between the levels were expressed as fold changes with the $2^{-ßßCT}$ method. **A.** Comparison of the relative MUC1 expression values in tumor tissues from sacrificed mice indifferent immunization groups. **B.** and **C.** Comparison of the relative CD3e and CD8a expression valuesin tumor tissues from sacrificed mice in different immunization groups. **D, E, F.** and **G.** Comparison of the relative expression of Th1-associated markers IFNγRβ, CCR2, IL-2Rα and CCR5 in sacrificed mice from different immunization groups. ($r = 3$, $n = 3$).

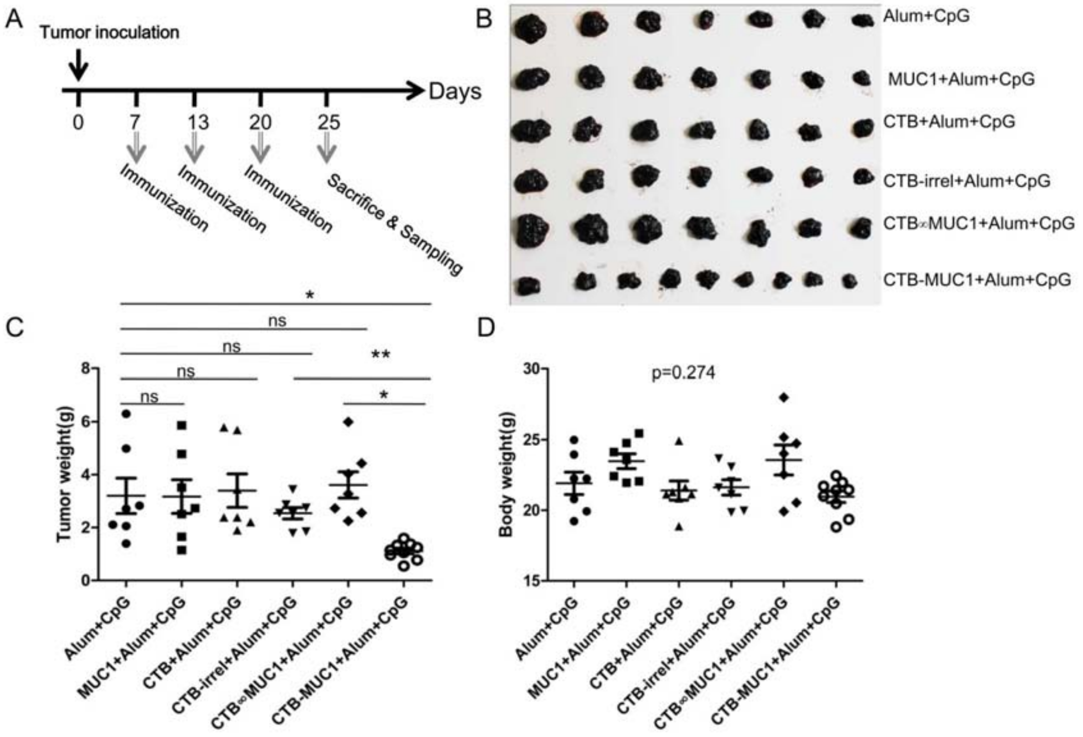

Supplementary Figure S4: Tumor therapeutic efficacy of CTB-MUC1 on MUC1⁺ B16 melanoma-bearing mice. **A.** Tumor therapy schedule. Mice got 3 immunizations from day 7 after tumor inoculation and about 1 vaccination per week. **B.** After sacrifice, tumors were removed from mice in different groups and photographed. **C.** Weight statistics of separated tumors after sacrifice. Each data point represents an individual mouse and the horizontal lines indicate the mean for the group of mice(n = 7–10). **D.** Mice body weight statistics on day 25. Statistical differences were analyzed by one-way ANOVA with Bonferroni's multiple comparison test.

Supplementary Table S1: Prediction of continuous B-Cell epitopes in CTB

Method	Method Parameters	Positions: Score of the Top 5 Prediction(peptide length ≥ 5AA)			
		1–20 Epitope*	30–50 Epitope	50–70 Epitope	70–103 Epitope
Chou and Fasman' sβ-turn scale	Window size 7<br>Minimum: 0.643<br>Maximum: 1.217	1–7: 1.13		53–59: 1.196<br>54–60: 1.183	88–94: 1.217<br>89–95: 1.174
Emini Surface Accessibility Prediction	Window size 6<br>Threshold: 1.000<br>Minimum: 0.136<br>Maximum: 3.587			58–63: 2.409<br>59–64: 3.672<br>66–71: 3.587	88–93: 2.690<br>89–94: 3.482
Bepipred Linear Epitope Prediction	Threshold: 0.350<br>Minimum:–0.925<br>Maximum:1.244	1–5	30–34	50–63	91–94
Kolaskar & Tongaonkar Antigenicity	Window size 7<br>Minimum: 0.915<br>Maximum: 1.163	5–12	47–60		72–80<br>82–89<br>93–99
Discotope - Prediction of epitopes from protein structure	Threshod: –7.7<br>PDB NO. 1CHQ		30–36	52–64	88–92

* The CTB protein sequence was divided into four parts to identify the location of continuous B-Cell epitopes

Supplementary Table S2: ELISA anti-MUC1 antibody subtypes and titers after 2 immunizations with various preparations

Immunization	IgG total	IgG1	IgG2a(IgG2c)	IgG2b	IgG3	IgM
CTB-MUC1+Alum	$100*2^{11}$	$100*2^{10}$	$100*2^{3}$	$100*2^{5}$	$100*2^{4}$	$100*2^{7}$
CTB-MUC1+Alum+CpG	$100*2^{12}$	$100*2^{11}$	$100*2^{6}$	$100*2^{10}$	$100*2^{6}$	$100*2^{5}$
CTB+Alum+CpG	$100*2^{2}$	100*2	<100*2	<100*2	<100*2	<100*2
MUC1+Alum+CpG	$100*2^{11}$	$100*2^{10}$	$100*2^{3}$	$100*2^{4}$	$100*2^{3}$	$100*2^{4}$
MUC1+Alum	$100*2^{5}$	$100*2^{4}$	100*2	$100*2^{3}$	$100*2^{2}$	$100*2^{2}$

Anti-MUC1 antibody titers are presented as median values for groups of 12–16 mice. ELISA plates were coated with $MUC1_{30}$ for anti-MUC1 antibody titers.Titers were determined by linear regression analysis, with plotting of dilution versus absorbance. Titers are defined as the highest dilution yielding an optical density of 0.1 or greater relative to normal control mouse sera.

Supplementary Table S3: Primers used in qPCR

Primer	Sequence(5′-3′)	Gene accessing number
mGAPDH-F	GCTAGGACTGGATAAGCAGGG	NM_008084.3
mGAPDH-R	AATCCGTTCACACCGACCTT	
hMUC1-VNTR-F	TCCGGCTCCGGGTTCTA	NM_001204285.1
hMUC1-VNTR-R	CAGAGCCGGACGGTTG	
mCD3e-F	CTGCTACACACCAGCCTCAA	NM_007648.4
mCD3e-R	CATCAGCAAGCCCAGAGTGA	
mCD8a-F	TCAGTTCTGTCGTGCCAGTC	NM_009915.2
mCD8a-R	TCACAGGCGAAGTCCAATCC	
mIL-2Rα-F	AACACCACCGATTTCTGGCT	NM_008367
mIL-2Rα-R	GCTGGCCACTGCTACCTTAT	
mCCR2-F	CAAGCACTTAGACCAGGCCA	NM_009915.2
mCCR2-R	ACTCGATCTGCTGTCTCCCT	
mIFN-γRβ-F	TCCTCGCCAGACTCGTTTTC	NM_008338.3
mIFN-γRβ-R	GGGTCATTGCTGGAAGGTGA	
mCCR5-F	GTATGTCAGCACCCTGCCAA	NM_009917.5
mCCR5-R	GCAGGAAGAGCAGGTCAGAG	

All the primers were designed to amplify a 100–200bp product unique to template. The Tm of the primers were set at 57°C–60°C
